# Serum Therapy for Diphtheria

**Published:** 1894-12

**Authors:** 


### *EDITORIAL.*

The office of THE JOURNAL is in room 330 Equitable Building.

Address all communications, and make all remittances payable to THE ATLANTA MEDICAL AND SURGICAL JOURNAL, Atlanta, Ga.

Articles for publication should reach this office not later than the 15th instant.

The editors are not responsible for views expressed by contributors.

### SERUM THERAPY FOR DIPHTHERIA.

Now for many months the most crucial tests have been made of the antitoxin treatment of diphtheria, with highly encouraging results.

For two or three years investigations and experiments have been going on in Germany, chiefly by Behring, Wasserman, Ehrlich, and Wernicke, to find, if possible, a specific remedy for diphtheria. The material employed has been the blood-serum of animals immune against the disease. A substance was isolated from the ptomaines of the diphtheria bacilli, to which the name "toxin" was given. Horses are inoculated with this toxin up to the point when the animal's system fails to respond to further inoculation. Blood is then drawn, and the serum collected—the antitoxin now in use. It is a clear, colorless liquid, of syrupy consistency, having an odor slightly resembling that of carbolic acid. The remedy is administered hypodermically, between the scapulæ, in the loins or thighs. The usual dose is five to ten cubic centimetres (1¼ to 2½ drams). No systemic reaction follows. One injection is sufficient for some cases; in others the treatment is repeated on the following day.

Outside of Germany, M. Roux, of Paris, has been the most active worker with the new remedy. Dr. Roux has been the assistant of

Pasteur for many years. He has given the greater part of this year to the investigation of antitoxin, and deserves great credit for improvements upon the original German methods of using the remedy.

Now as to results. In the Children's Hospital of Berlin, for the five years preceding the introduction of the antitoxin treatment in March last, the annual mortality from diphtheria had averaged forty per centum. Between March 14th and June 20th, one hundred and twenty-eight cases were treated with antitoxin, with a mortality of 16.5 per cent., as compared with a mortality of thirty-seven per cent. for the same period last year. Roux relates that in one of the hospitals of Paris the total mortality in the diphtheria wards for five years was 51.7 per cent.; while under the antitoxin treatment from February 1 to July 25, 1894, the mortality was 24.5 per cent. During the same time cases of diphtheria at the Trousseau Hospital, treated without the antitoxin, had a mortality of sixty per cent. In cases of simple diphtheria, not requiring tracheotomy, the mortality for four years has been 33.9 per cent. Under the antitoxin treatment at *l'Hospital des Enfants* the mortality fell to twelve per cent., while it was thirty-two per cent. at the Trousseau Hospital (no antitoxin) during the same period. In cases requiring tracheotomy the mortality for four years has been seventy-three per cent.; with the new treatment the mortality for similar cases fell to forty-nine per cent. At the Trousseau Hospital (no antitoxin) the mortality has been eighty-six per cent. Other reports have been made which correspond pretty closely with the above. Thirty cases reported by Behring and Kossell had a mortality of twenty per cent. Ehrlich and Wasserman reported sixty-seven cases with tracheotomy, with a mortality of 44.9 per cent.; and one hundred and fifty-three cases without tracheotomy, with a mortality of 23.6 per cent. Katz reports one hundred and twenty-eight cases, mortality 13.2 per cent.; Aronson, one hundred and ninety-two cases, mortality thirteen per cent.; Roux, four hundred and forty-eight cases, mortality 24.3 per cent. More than one thousand cases reported show an average mortality of twenty-four per cent. In one London hospital the mortality now stands at ten per cent. These figures apply only to cases which were known to have been true diphtheria by the presence of the Klebs-Loeffler bacillus.

Very little has been done with the new treatment in America. The last issue of the *Medical News* (November 17) publishes the results of some studies by Dr. W. M. Welch, of the Municipal Hospital, Philadelphia. Five cases are reported, with one death. In the Willard Parker Hospital, New York, twenty severe cases have been subjected to this treatment. In six cases the disease was confined to the fauces; one died of scarlatina, developed during convalescence from the diphtheria. Fourteen cases had also laryngeal diphtheria; among these the mortality was 28.5 per cent.

Such is the present status of the antitoxin or serum treatment of diphtheria. The figures and facts presented certainly encourage us to hope that we now have at hand a remedy which may prove a conqueror of this dread disease.

The British *Medical Journal* (November 10th) says: "The results achieved both in this country and abroad no longer leave room for doubt of the very high value of this remedy as a curative means." Dr. Louis Fischer, of New York, says (*Medical Record*, October 6): "If we can rely on statistics, we have in antitoxin not only a positive remedy in diphtheria, but the beginning of a new form of treatment which will eventually save the lives of thousands of patients."

The matter was much discussed at the late meeting of the Hygienic Congress at Buda-Pest, attended by more than two thousand European physicians and scientists. Many of those present regarded antitoxin as a remedy of great promise, both prophylactic and curative. We earnestly hope that the history of tuberculin a few years ago will not repeat itself with the new treament for diphtheria.

